# Rectourethral Fistula Secondary to Transurethral Resection of the Prostate

**DOI:** 10.7759/cureus.3476

**Published:** 2018-10-22

**Authors:** Vijay Gayam, Amrendra Mandal, Pavani Garlapati, Mazin Khalid, Binav Shrestha, Arshpal Gill

**Affiliations:** 1 Internal Medicine, Interfaith Medical Center, Brooklyn, USA

**Keywords:** transurethral resection of the prostate, rectourethral fistula, benign prostate hyperplasia

## Abstract

An 82-year-old male with benign prostatic hyperplasia (BPH) who underwent transurethral resection of the prostate (TURP) presented to the hospital with suprapubic pain, abdominal distension, and diarrhea. The physical examination was remarkable for an indwelling Foley’s catheter. Diagnostic imaging confirmed the diagnosis of a rectourethral fistula (RUF). The most common presenting symptoms of RUF are pneumaturia, fecaluria, and urine leakage from the rectum, which may present similarly to diarrhea. He lacked the common features of RUF such as pneumaturia and fecaluria, which may be explained by a blockage of the catheter with fecal material. This case represents a rare outcome following a TURP, and it is significant due to the high morbidity associated with RUF. As such, clinicians must suspect a RUF in a post-TURP patient with diarrhea and no other obvious etiology due to the morbidity associated with RUF.

## Introduction

Benign prostatic hyperplasia (BPH) is becoming more common in the aging male population. The transurethral resection of the prostate (TURP) is the mainstay of treatment for BPH and is currently the standard of care. Rectourethral fistula (RUF) represents a pathological communication between the rectum and urinary tract. A RUF is extremely uncommon, with an incidence of 0.5 per 100,000 per year [1]. RUF can occur following trauma, inflammatory bowel disease, urological procedures, colorectal surgeries, and other rare causes [1 ]. Interventions such as radical prostatectomy, radiotherapy (RT), TURP, brachytherapy (BT), etc. may lead to RUF [2]. The diagnosis of RUF can be made with the following investigations: cystourethroscopy, colonoscopy, and a contrast study of the rectum or CT scan of the abdomen and pelvis [3]. Surgical intervention remains the best treatment option. However, surgical repair of RUF is challenging without any standardized approach [2]. This article focuses on the unusual complication of TURP as well as its atypical presentations after the development of RUF.

## Case presentation

**Figure 1:**
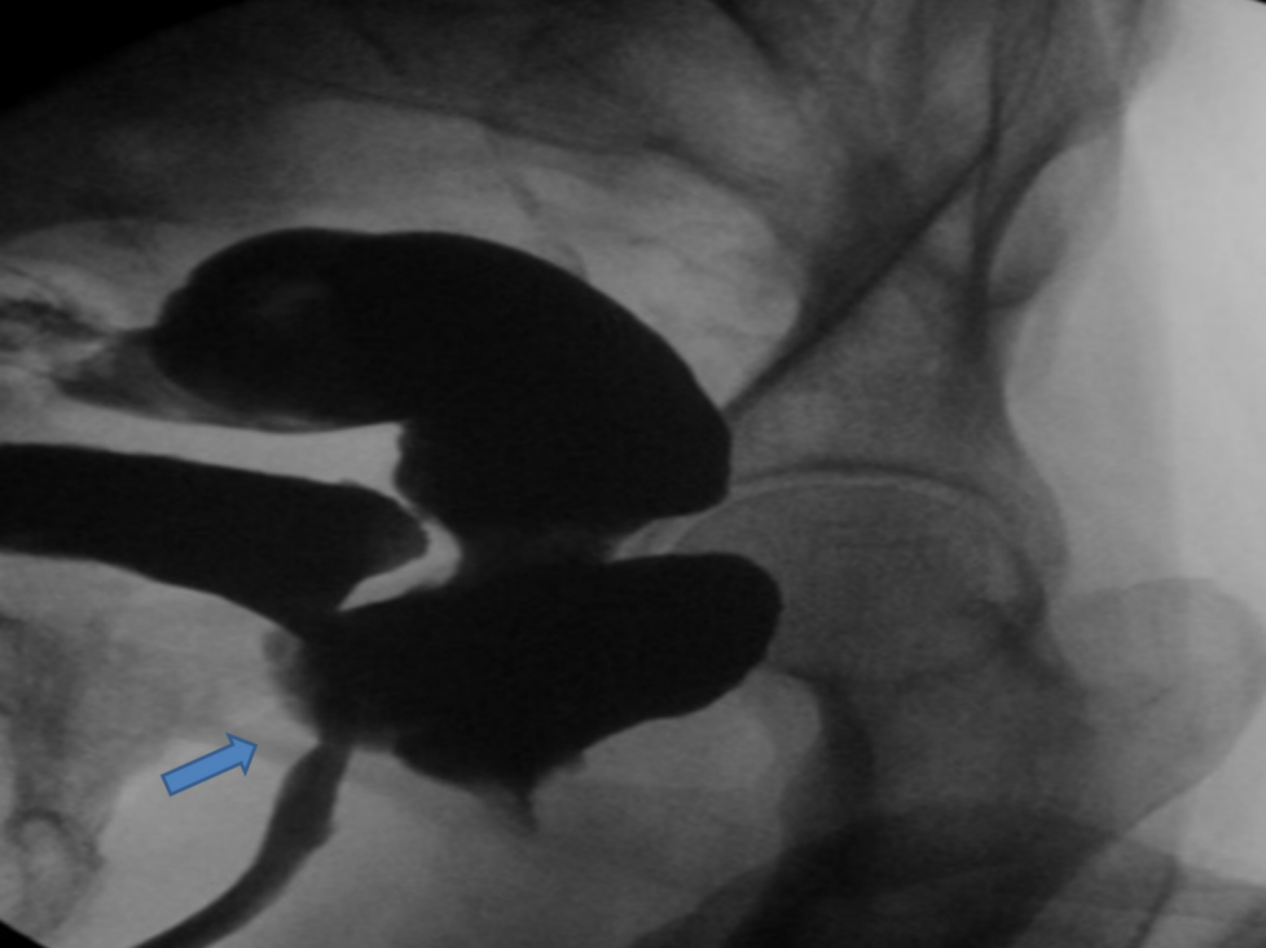
Cystourethrography revealing a rectourethral fistula Blue arrow shows the rectourethral fistula

An 82-year-old male with a history of type 2 diabetes mellitus, hypertension, benign prostatic hyperplasia (BPH) with a size of 40 gram presented with asthenia, suprapubic pain, and distension of the lower abdomen. He underwent transurethral resection of prostate (TURP) for his BPH one week prior to admission and had an indwelling Foley’s catheter (due to urinary incontinence) at presentation. He also complained of non-bloody, watery diarrhea with four to five episodes per day for five days. He denied fever, cloudy urine, purulent urethral secretion, or any back pain. He also denied any prior history of radiotherapy or other gastrointestinal (GI) surgery related to the colon. At presentation, the patient was alert, awake, and cooperative and his vital signs included a temperature of 98.6° F, respiratory rate of 14 per minute, pulse of 86 beats per minute, BP of 113/56 mm Hg, and saturating 99% in room air. The physical examination revealed an indwelling Foley catheter with an attached right thigh bag showing clear urine. Laboratory investigations were notable for a slight elevation of the creatinine from the baseline. The urinalysis was positive for leukocyte esterase and nitrates, five to 15 red blood cell/high power field (HPF), and 30-50 white blood cell/HPF. The stool culture revealed no growth of microorganisms. The patient was started on intravenous (IV) normal saline and IV ceftriaxone 1 gram daily for a suspected urinary tract infection. Computed tomography (CT) of the abdomen and pelvis with IV contrast demonstrated findings consistent with a RUF. Cystourethrogram under fluoroscopy showed the extravasation of contrast into the rectum, which is also consistent with a rectourethral fistula (RUF) (Figure 1).

**Figure 2:**
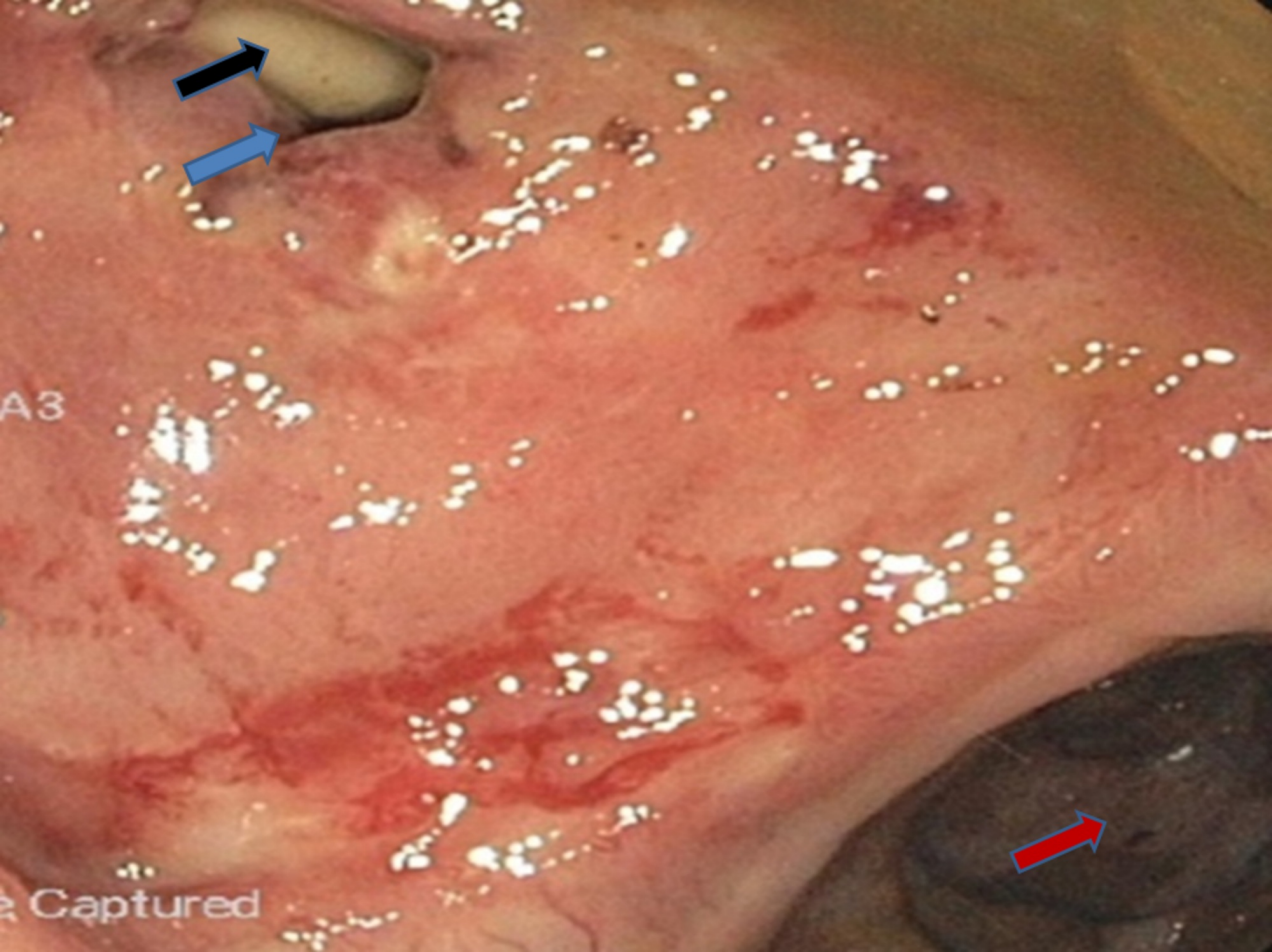
Colonoscopy revealing the catheter in the rectourethral fistula and partially present in the rectum Blue arrow shows the rectourethral fistula Black arrow shows Foley's catheter Red arrow shows the rectosigmoid junction

**Figure 3:**
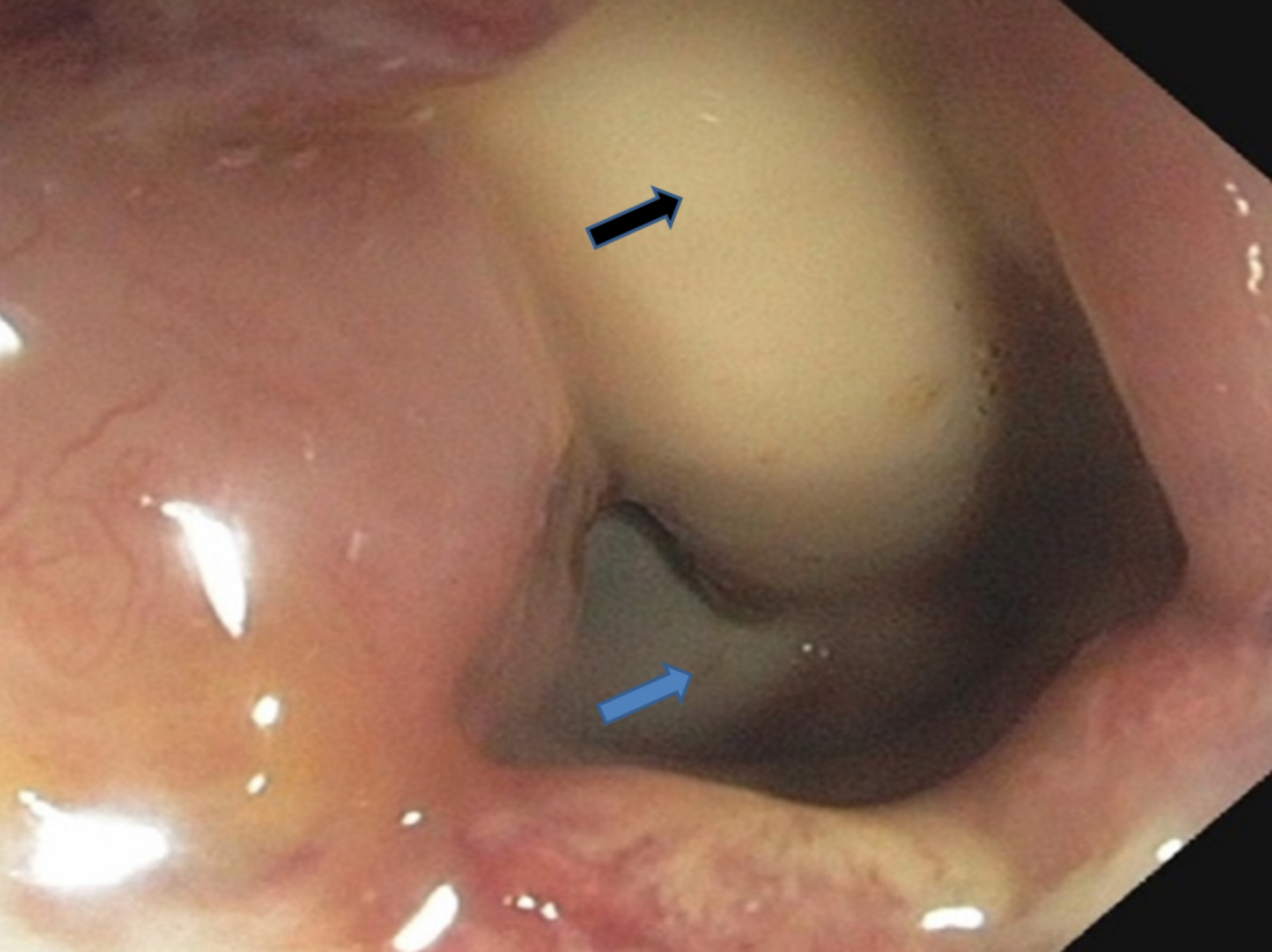
Closer view of the rectourethral fistula with Foley's catheter Blue arrow shows the rectourethral fistula Black arrow shows Foley's catheter

A colonoscopy was performed and revealed a Foley's catheter in the rectum (Figures 2-3). A rectal biopsy was not obtained, as, grossly, there was no evidence of malignancy, infectious process, or inflammatory process.

**Figure 4:**
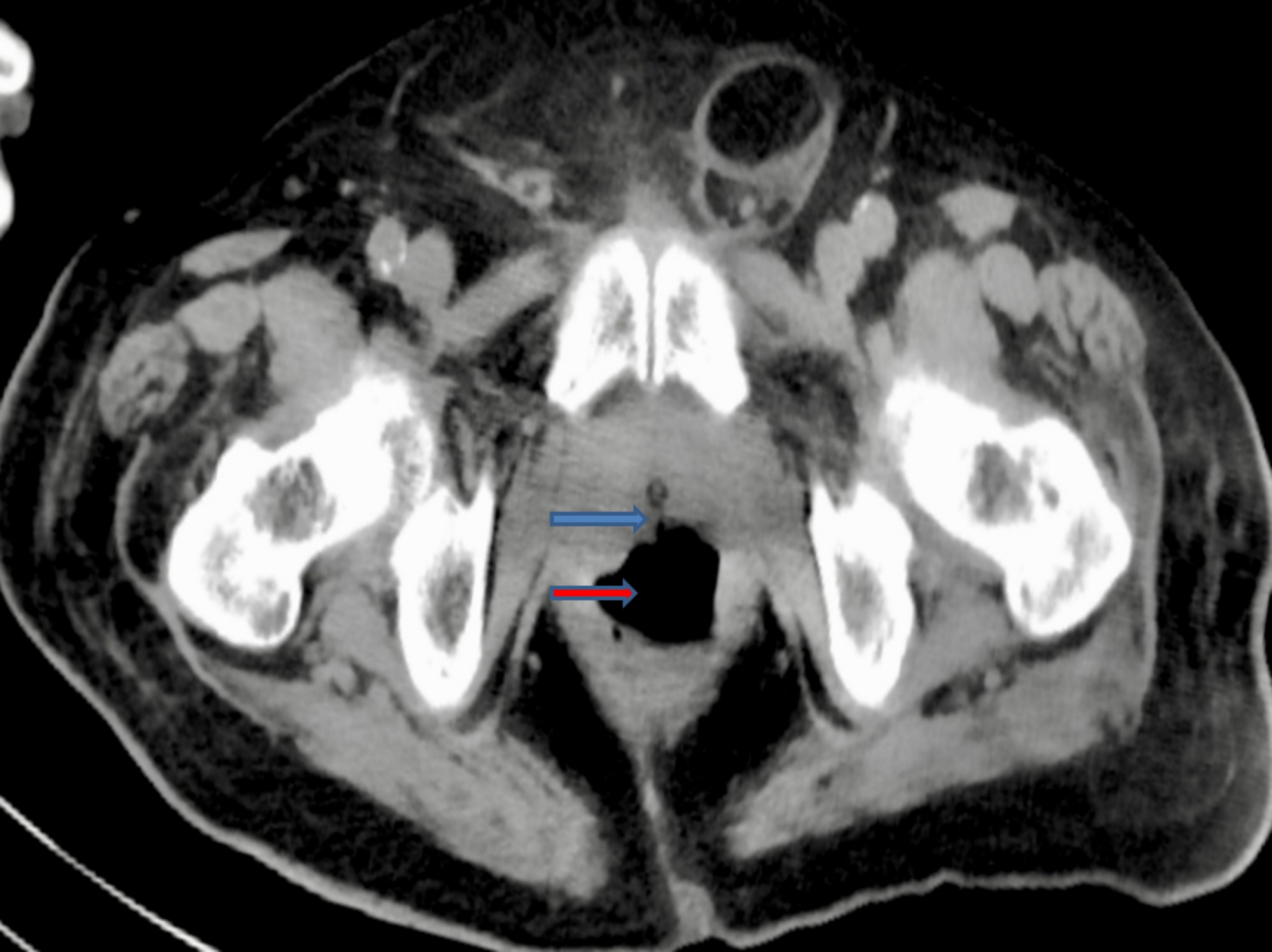
Computed tomography (CT) abdomen and pelvis revealed the rectourethral fistula Blue arrow shows the rectourethral fistula Red arrow shows the rectum

**Figure 5:**
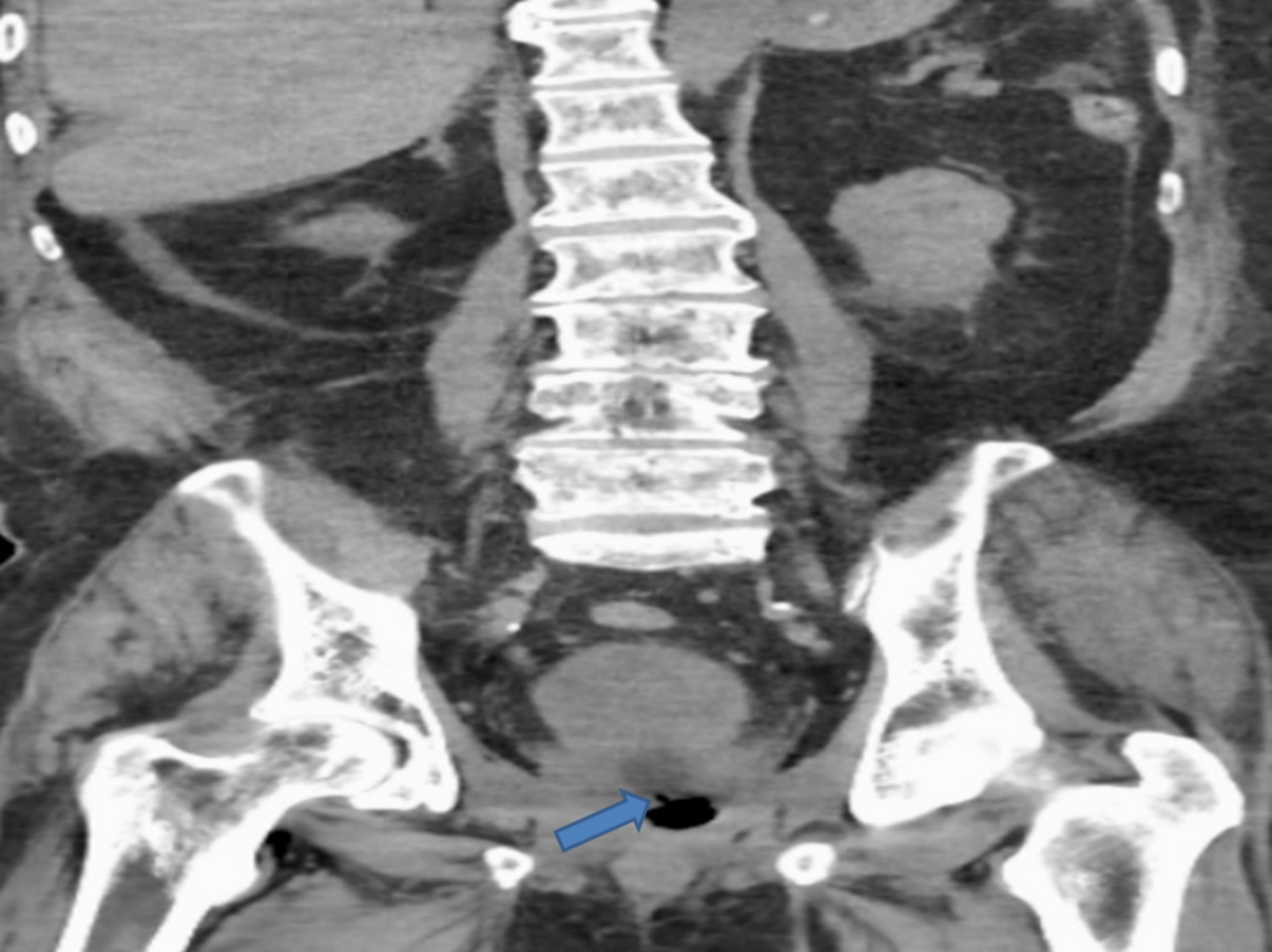
Computed tomography (CT) abdomen and pelvis revealed the rectourethral fistula Blue arrow shows the rectourethral fistula

The abdominal and pelvic CT scan also showed RUF (Figures 4-5).

## Discussion

A rectourethral fistula is an uncommon disorder but is associated with significant morbidity and mortality. TURP represents an unusual cause of RUF. The symptoms of RUF include leakage of the urine from the rectum, pneumaturia, and fecaluria, and a RUF is associated with severe co-morbidities [3]. Our patient’s initial complaint was watery diarrhea, which is atypical for RUF. The more common symptoms, fecaluria and pneumaturia, were likely absent due to the malposition and blockage of the catheter with the fecal material. RUF is initially best investigated with an anterograde and retrograde urethrogram, but these may lack sensitivity for a smaller RUF. More enhanced imaging techniques have better sensitivity, such as the direct visualization of the fistulous opening via cystoscopy. As a result, cystoscopy is the gold standard of imagining for RUF with a sensitivity of 80% to 100% [4]. The management of RUF varies, with conservative and surgical therapies both being viable options based on the patient’s condition and local availability of urological expertise. Consequently, there is no consensus in for a gold standard of care for these fistulae [5]. The transperineal repair approach, with pedicled gracilis muscle interposition, was regarded as the most favorable approach in one study involving 53 patients [6]. A laparoscopic repair of RUF is an appealing alternative surgical approach. High operative risk patients may be managed with non-surgical methods, including endoscopic injection of fibrin glue for non-malignant RUF and the application of covered colonic stents [7-8].

## Conclusions

TURP is a routinely done procedure for BPH that very rarely has RUF as a complication. In these extreme circumstances, the morbidity rate is high. As a result, clinicians must have a low index of suspicion in a post-TURP patient with concurrent symptoms of RUF, including urinary leakage from the rectum, pneumaturia, and fecaluria.
